# Supplementary material for: Health facility readiness to provide antenatal care (ANC) and non-communicable disease (NCD) services in Nepal and Bangladesh: Analysis of facility-based surveys
Source: PLoS One. 2023 Mar 13;18(3):e0281357. doi: 10.1371/journal.pone.0281357 (PMC10010536; doi:10.1371/journal.pone.0281357)
Supplement: S1 Table — (DOCX) [file pone.0281357.s001.docx]

**S1 Table. Factors associated with readiness for providing only ANC services (binary logistic regression estimating ORs).**

| **Variables** | **Nepal (N = 1565)** | | **Bangladesh (N = 512)** | |
| --- | --- | --- | --- | --- |
|  | **Unadjusted OR (95%CI)** | **Adjusted OR (95%CI)** | **Unadjusted OR (95%CI)** | **Adjusted OR (95%CI)** |
| **Managing authority** |  |  |  |  |
| Public | Ref. | Ref. | Ref. | Ref. |
| Private/NGO | 5.7*** (3.9-8.4) | 6.0***(3.9-9.2) | 8.2***(4.5-14.8) | 3.5***(2.1-6.0) |
| **Location** |  |  |  |  |
| Rural | Ref. | Ref. | Ref. | Ref. |
| Urban | 1.1 (0.8-1.5) | 1.0 (0.7-1.3) | 6.7***(4.0-11.3) | 2.2** (1.4-3.5) |
| **Routine quality assurance** |  |  |  |  |
| Not performed | Ref. | Ref. | Ref. | Ref. |
| Performed | 2.0** (1.3-3.0) | 1.9** (1.3-2.9) | 4.4***(2.6-7.5) | 2.9*** (1.7-5.1) |
| **System to obtain client feedback** |  |  |  |  |
| No | Ref. | Ref. | Ref. | Ref. |
| Yes | 2.0***(1.5-2.8) | 1.8***(1.3-2.5) | 3.4***(2.4-4.8) | 1.9** (1.3-2.8) |
| **External supervision in the previous 4 months** |  |  |  |  |
| Did not occur | Ref. | Ref. | Ref. | Ref. |
| Occurred | 1.5* (1.1-2.0) | 1.5* (1.1-2.1) | 1.2 (0.7-1.9) | 1.4 (0.8-2.5) |
| **Regular monthly management meetings** |  |  |  |  |
| No | Ref. | Ref. | - | - |
| Yes | 1.3^*^ (1.0-1.8) | 1.1 (0.76-1.5) | - | - |
| **P*<0.05, ***P*<0.01, ****P*<0.001, - variables not reported | | | | |
