# Supplementary material for: Health facility readiness to provide antenatal care (ANC) and non-communicable disease (NCD) services in Nepal and Bangladesh: Analysis of facility-based surveys
Source: PLoS One. 2023 Mar 13;18(3):e0281357. doi: 10.1371/journal.pone.0281357 (PMC10010536; doi:10.1371/journal.pone.0281357)
Supplement: Attachment — Submitted filename: Response to Reviewers 24 June 2022.docx [file pone.0281357.s002.docx]

24 June 2022

Natasha McDonald, PhD

Associate Editor

*PLOS ONE*

Re: *Revision PONE-D-21-26082 Health facility readiness to provide antenatal care (ANC) and non-communicable disease (NCD) services in Nepal and Bangladesh: Analysis of facility-based surveys*

Dear Dr Natasha McDonald,

Thank you for your email dated 27 April 2022 suggesting some changes to the above manuscript. The comments made by the reviewers were very helpful and we have addressed all changes as notated in the Table below. A marked-up copy of the manuscript that highlights changes made to the original version (Revised Manuscript with Track Changes), and an unmarked version without tracked changes (Manuscript) has been submitted.

We would like to express our thanks for providing the opportunity to incorporate the changes and hope that the revised manuscript meets with your approval.

Thank you for your consideration and we look forward to hearing from you in due course.

Sincerely,

Dr. Deependra Kaji Thapa (on the behalf of all authors)

Public Health Research and Development Center

Kathmandu, Nepal

[thapa.deepen@gmail.com](mailto:thapa.deepen@gmail.com)

**Response to the editor/reviewers’ comments**

**Manuscript ID PONE-D-21-26082**

**Health facility readiness to provide antenatal care (ANC) and non-communicable disease (NCD) services in Nepal and Bangladesh: Analysis of facility-based surveys**

| **Comments** | **Response** |
| --- | --- |
| **Editor** |  |
| The reviewer raised a number of major and minor concerns that must be addressed. They felt that the research question was not presented clearly and therefore the rationale of the study was not fully evident. They also felt that aspects of the methodology were not presented clearly. Their comments can be viewed in full, below. | Thank you for the opportunity to revise the manuscript. Comments and suggestions have been addressed as detailed below.  We also thank the reviewers for their positive and encouraging comments and suggestions. These were very helpful and have improved the quality of our paper. We have addressed all comments, amending the manuscript as suggested. |
| **Reviewer’s Comments:** |  |
| **Major** |  |
| 1) Conceptual framing. Two issues: what is the value of analyzing multiple countries given the differences in population needs, health system design, and policymaking process? Given for instance the decentralized approach to health service administration in Nepal, what is the value to a district policymaker of seeing data from Nepal compared to Bangladesh and Afghanistan (particularly since, as noted below, the ASPA is quite difficult to compare to any other)? Including multiple countries makes it more difficult to provide a nuanced analysis and policy recommendations specific to one setting, so there should be some clear justification for the value of doing so. | Thank you for highlighting this concern. We have amended the manuscript and removed Afghanistan from the analysis. However, both Nepal and Bangladesh have similar health systems, with shared challenges common to low- and middle-income countries. The added value of including these two countries and justification for doing so is now included in the paragraph prior to the Method section of the manuscript. |
| Secondly, the concept of integration is tangential to the analysis here, which can speak only to co-occurence of readiness for ANC and NCDs. Readiness for each service does not imply ready to integrate, as the latter requires shared knowledge, space, resources, patient flow, etc.  Further, the introduction sets up the analysis more towards the question, 'Of facilities offering ANC, how many might be prepared to provide NCD care if for instance policies focused on NCD services during ANC were pursued?' The analysis itself addresses the question, 'how many facilities meet bare minimum criteria to provide ANC and NCD services?', which is slightly different. I would suggest pulling back the talk of integration and revising the denominator to focus on the more narrow and more actionable question of NCD readiness within ANC facilities. The conceptual basis for the explanatory variables tested in the multivariable regression requires further detail. | Thank you for raising this important observation. Our operational definition for service integration is co-occurrence of readiness for ANC and NCDs. In line with the reviewer’s observation, we have re-named the outcome variable from ‘service integration readiness’ to ‘facility readiness to provide both ANC and NCD services’. We have also removed the word ‘integration’ from the title of the manuscript.  The introduction sets up on the opportunity of integrating ANC and NCD services. We aimed to assess readiness for providing ANC and NCD services, which has been made more explicit in our study aim. The introduction section has been revised providing more focus on the importance of readiness to provide both ANC and NCD services. In terms of analysis, previously we have included only those facilities which provided either ANC or NCD services. In the revised version we re-analysed the data including all the health facilities except the stand-alone HIV testing and counselling centres (HTCs) and community clinics which are not expected to provide ANC and/or NCD services. Furthermore, we have also included analysis on facilities showing NCD service readiness among those facilities which were offering any ANC service (Fig 1) (Note: Most of the facilities were providing ANC services). |
| 2) Methods: inclusion of ASPA. The Afghanistan SPA has a different target population than all other SPA, and as such provides limited insight in a comparative analysis. At most it should be compared only to hospitals in other settings. The description of the ASPA including hospitals "in addition" is misleading - in fact the whole survey is focused on hospitals / referral centers in urban areas. | We agree and have deleted “in addition” and Afghanistan accordingly. Thank you for raising this. |
| **Minor** |  |
| Where did staff training items come from, provider interviews? Operationalized as any respondent indicating relevant training? In results section, it sounds as if the same provider had to be trained in ANC and NCDs, while in the methods section it sounded like this was assessed separately by service (e.g., at least one provider trained in ANC, at least one provider trained in NCDs, whether or not they are the same person). Similarly the laboratory items in SPA are not specific to ANC and NCDs, please clarify | Staff training items were assessed by provider interview, defined as “At least one staff member trained in ANC (for ANC), and diabetes, CVD and CRD diagnosis and treatment (for NCD)”. As we undertook the facility level analysis there could be different health workers trained in ANC or NCDs.  The diagnostics domain under the ANC service readiness measured services for Hemoglobin (Hb) and Urine (dipstick- protein). Under the NCDs they were Blood glucose, Urine protein, and Urine glucose which were as per the SPA items for service readiness. |
| Wide range in CIs in Table 5 suggests sparse data. | The data has been re-analysed, and addressed the issues identified in the previous version of the manuscript. |
| Please note if/that sampling weights were used when describing incorporation of survey attributes | Sampling weights were used to correct for non-responses and disproportionate sampling. This has been specified in the data analysis section under methodology. |
| What data source was used to check whether Nepal facilities were urban or rural based on the GPS coordinates? | Location of the facilities (rural and urban) were classified according to the administrative units based on the available GPS coordinates and defined rural (rural municipality) and urban areas (metropolitan/sub metropolitan city and municipality). This has been clarified in the manuscript. |
