# Supplementary material for: Health facility readiness to provide antenatal care (ANC) and non-communicable disease (NCD) services in Nepal and Bangladesh: Analysis of facility-based surveys
Source: PLoS One. 2023 Mar 13;18(3):e0281357. doi: 10.1371/journal.pone.0281357 (PMC10010536; doi:10.1371/journal.pone.0281357)
Supplement: Attachment — Submitted filename: Response to reviewer 8 Nov 2022.docx [file pone.0281357.s003.docx]

**Response to the editor/reviewers’ comments**

**Manuscript ID PONE-D-21-26082R1**

**Health facility readiness to provide antenatal care (ANC) and non-communicable disease (NCD) services in Nepal and Bangladesh: Analysis of facility-based surveys**

| **Comments** | **Response** |
| --- | --- |
| **Editor** |  |
| Please note the reviewer's suggestion of comparing facilities offering ANC and NCD services to those who can offer neither or only one as a way of providing greater insight on what determines joint readiness. The reviewer aptly notes that the current findings are not substantially different from availability and readiness of NCD services alone given the broad availability of ANC services. Tabular presentation of this 4-group comparison would enrich the study and provide more substance to this work; the comparison could be repeated considering readiness as well as availability. | Thank you for the opportunity to revise the manuscript.  This paper assesses readiness of health facilities to provide both ANC and NCD services, not the availability of both services. Please note that although most of the facilities (>95%) are offering ANC services, the readiness score for ANC is only 53% in Nepal and 54% in Bangladesh.  As suggested, we have included a 4-group comparison (only ANC, only NCD, both ANC and NCD, and neither) for availability, and readiness. |
| **Reviewer’s Comments:** |  |
| Thank you for the opportunity to review this manuscript. It investigates a topic of importance: to what extent are health facilities in LMICs (here, Bangladesh and Nepal) ready to offer NCD services for women seeking ANC. | Many thanks for your comments. This paper assesses readiness of health facilities to provide both ANC and NCD services.  We have addressed comments as below. |
| I do not feel the analysis offers an advance beyond what we already know, namely that very few facilities offer and/or are ready to provide NCD services. The “combined” availability and readiness numbers are presumably heavily driven by the NCD numbers (they parallel them quite closely). | Thank you for highlighting this concern. We have updated 2015 data for Nepal with the recently released 2021 health facility survey. The results shows that 72% of health facilities in Nepal (2021) and 35% in Bangladesh (2017) have availability of NCD service, while the NCD readiness is 28.4% and 18.2% respectively in these countries. |
| To my understanding the authors did not look at “types” of facilities, i.e. can offer ANC only, can offer NCD only, can offer both, can offer neither – this possibly could have highlighted something new if, for example, the facilities offering ANC+NCD are a unique subset of those offering NCD-only. | Many thanks for this suggestion. We have added 4-group comparison (only ANC, only NCD, both ANC and NCD, and neither) for availability, and readiness. |
| These data have been analyzed in numerous papers making a similar point about NCD services, and the data are quite old at this point (5 and 7 years). The paper is well-written and clear but I just do not feel that it offers anything that has not already been well-documented previously. | We have replaced the 2015 data from Nepal with the recently released 2021 Health Facility Survey in support of the contribution this paper we believe makes. |
