# Supplementary material for: Health facility readiness to provide antenatal care (ANC) and non-communicable disease (NCD) services in Nepal and Bangladesh: Analysis of facility-based surveys
Source: PLoS One. 2023 Mar 13;18(3):e0281357. doi: 10.1371/journal.pone.0281357 (PMC10010536; doi:10.1371/journal.pone.0281357)
Supplement: Attachment — Submitted filename: Response to Editor 30 Dec 2022.docx [file pone.0281357.s004.docx]

**Response to the editor/reviewers’ comments**

**Manuscript ID PONE-D-21-26082R2**

**Health facility readiness to provide antenatal care (ANC) and non-communicable disease (NCD) services in Nepal and Bangladesh: Analysis of facility-based surveys**

| **Comments** | **Response** |
| --- | --- |
| **Editor** |  |
| I appreciate the careful revision and the decision to update the article to reflect the latest Nepal data. I would make several minor suggestions: | Thank you for your supportive comments, and the opportunity to revise the manuscript.  We have addressed the comments as detailed below. |
| - revise wording of 'willingness to provide' as 'capacity' since readiness is about tangible capacity, not provider motivation / interest in providing a service | Revised as suggested. |
| - replace 'weightage' with weight or weighting | Thank you, we have changed ‘weightage’ to ‘weighting’ as suggested. |
| - remove 'integration' from results section wording (consider 'joint readiness') and revisit in discussion how the analysis of readiness for both ANC and NCD speaks to the basis that facilities have for providing integrated services. | The word ‘integration’ in the Results section has been replaced with ‘joint readiness’.  We have revised the Discussion section (on policy implications) and included how the analysis of readiness for both ANC and NCD speaks to the basis that facilities have for providing integrated services.  *The analysis of readiness for both ANC and NCD services provides a basis that facilities in LMICs have in providing integrated services. For successful integration of ANC and NCD services, health planners and policymakers should ensure adequate availability of skilled personnel, policy, guidelines and standards, and diagnostics, medicines, and commodities. Evidence provided in this study highlights the gap in joint readiness for integrating ANC and NCD services and the need to strengthen capacity in both Nepal and Bangladesh. .* |
| It would be interesting to see if the facilities that are ready for both services differ from facilities ready for ANC only in each country, and if the factors predicting this joint vs. single readiness are similar to those distinguishing joint readiness from all other facilities. This is to the authors' consideration, and not a requirement for further consideration. | Thanks for this comment.  We analyzed whether the facilities that are ready for providing both ANC and NCD services differ from facilities ready for ANC only in each country. The findings are presented in the Supplementary file 1 and are also described in the Results section.  *In addition, we assessed whether the facilities that are ready for providing both ANC and NCD services differ from facilities that are ready for ANC only in each country. Most of the factors predicting readiness for only ANC services were similar to those predicting joint readiness in both countries (Supplementary file 1). Health facilities managed by the private sector or an NGO, facilities undertaking routine quality assurance, and having a system to obtain client feedback were positively associated with service readiness for providing only ANC services. Differences were observed in external supervision and regular monthly management meetings. Facilities supervised in the previous 4 months had higher odds for ANC only readiness index, while regular monthly management meetings were not associated with ANC only service readiness in Nepal.* |
| **Reviewer’s Comments: None** | Thank you. No action required. |
